# Effects of personalized nursing on treatment adherence and clinical symptoms in prostatitis patients

**DOI:** 10.3389/fmed.2025.1672376

**Published:** 2025-10-15

**Authors:** Yueting Zhu

**Affiliations:** Department of Urology Ward One, The First Affiliated Hospital of Harbin Medical University, Harbin, China

**Keywords:** personalized nursing, prostatitis, treatment adherence, inflammatory factors, negative emotions, health behaviors, urinary flow rate, quality of life

## Abstract

**Background:**

Chronic prostatitis is a common urological condition, often presenting with urinary irritation and chronic pelvic pain. These symptoms can negatively affect patients’ daily life and treatment adherence, with some showing poor cooperation during therapy. Therefore, appropriate nursing guidance is essential to ensure treatment efficacy and support self-management.

**Objective:**

This study aimed to evaluate the impact of personalized nursing on treatment adherence and clinical symptoms in patients with prostatitis.

**Methods:**

Eighty-five prostatitis patients were enrolled and randomly separated into two groups. The control group (*n* = 47) adopted conventional nursing mode; the observation group (*n* = 38) adopted personalized nursing mode on top of the control group. Before and after nursing, the clinical symptoms were assessed by the National Institutes of Health-Chronic Prostatitis Symptom Index (NIH-CPSI); the psychological status was evaluated by the Self-rating Anxiety Scale (SAS) and the Self-rating Depression Scale (SDS). The maximum and average urine flow rate, the treatment adherence, and the health behavior competence, including health responsibility, nutrition, exercise, and psychological well-being were compared. Prostate fluid specimens were collected and the levels of tumor necrosis factor-*α* (TNF-α), prostaglandin E2 (PGE_2_) and cyclooxygenase-2 (COX-2) were measured by radioimmunoassay.

**Results:**

Following nursing intervention, both groups showed improvements across clinical and biochemical parameters. NIH-CPSI, SAS, and SDS scores declined significantly, while maximum and average urinary flow rates increased. Treatment adherence and health behavior competence were also enhanced in both groups. Notably, the observation group demonstrated greater improvements in all these outcomes, with lower symptom and psychological scores, higher urinary flow rates, better adherence and self-management, as well as more pronounced reductions in TNF-*α*, PGE_2_, and COX-2 levels compared with the control group.

**Conclusion:**

Personalized nursing improves treatment adherence, health behaviors, and quality of life in patients with prostatitis.

## Introduction

Prostatitis, defined as pathological inflammatory changes in the prostate (1), is a prevalent urinary infectious disorder (2). Prostatitis can result in frequent urination and pelvic pain, and long-term prostatitis can raise the risk of benign prostate hyperplasia and prostate cancer (3). Prostatitis, a major urological disease, can influence 25–50% of men throughout their lifetime (4). Prostatitis is problematic to be treated effectively and generates complains about the genital and urinary systems globally (5). Beyond physical symptoms, chronic prostatitis/chronic pelvic pain syndrome (CP/CPPS) substantially impairs quality of life and psychological well-being, with an impact comparable to other chronic conditions (6). Psychological factors, particularly pain catastrophizing and emotional distress, are highly prevalent among CP/CPPS patients and strongly correlate with symptom severity (7). Such evidence highlights the importance of multidimensional management strategies.

Given the high prevalence, chronicity, and multidimensional impact of prostatitis, effective management requires not only medical therapy but also comprehensive nursing engagement. With the continuous advancement of urology as a specialty, the role of nursing has expanded beyond traditional bedside care to encompass specialized procedures, multidisciplinary coordination, and long-term patient management. Nurses are taking on roles and activities such as prostate biopsy procedures, and urology nursing has gradually developed as a specialty in the past few decades (8). Clinical research nurses play a vital role in initial contacts, monitoring and follow-up because of their high levels of patient contact (9). Recent evidence suggests that urology clinical nurse specialists, as part of multidisciplinary teams (MDTs), not only provide technical support but also advocate for patients’ psychosocial needs, although their voices are sometimes underrepresented in MDT meetings (10). A annualized and standardized intervention in primary care can sustainably reduce lower urinary tract symptoms in men (11). In addition, accelerated rehabilitation nursing (ARN), also known as enhanced recovery after surgery (ERAS), has been demonstrated to improve recovery outcomes in prostate cancer patients, including lower complication rates, shorter recovery time, better psychological well-being, and improved quality of life (12). Continuous nursing care in combination with cognitive behavioral intervention has been reported to substantially promote psychological status, self-care ability, and living quality while reducing the incidence of complications among patients after ureterolithiasis surgery (13). Furthermore, supportive nursing interventions and structured self-management programs play an essential role in addressing long-term quality-of-life concerns and unmet supportive care needs in prostate cancer survivors (14). Several diet and lifestyle factors are demonstrated to have an association with CP/CPPS and pain severity. Moreover, these modifiable conditions can be utilized as potential targets in the treatment of CP/CPPS (15) Individuals and family members engaged in self-management behaviors can improve their health results (16).

In this regard, personalized nursing interventions emphasize tailoring care to patients’ specific symptoms, psychosocial context, and lifestyle. This approach aligns with the UPOINT framework, first proposed by Shoskes et al. (17), which classifies CP/CPPS into six domains to guide individualized, multimodal management. Evidence further shows that phenotype-directed, UPOINT-guided therapy can reduce NIH-CPSI scores and improve patient outcomes, with benefits replicated in prospective cohorts (18). Validation studies in Chinese populations also support the clinical utility of UPOINT for phenotyping and management (19). These findings collectively support the rationale for investigating individualized nursing approaches in prostatitis patients, integrating physical, psychological, and behavioral dimensions to optimize outcomes.

Despite these advances, there remains limited evidence on the application of personalized nursing interventions in patients with prostatitis. Most existing studies have focused on surgical or oncological populations, while few have systematically evaluated individualized nursing strategies targeting treatment adherence, behavior modification, and symptom improvement in prostatitis. Personalized nursing—emphasizing tailored interventions based on patient-specific needs and psychosocial context—may provide a feasible and effective approach for this population. Therefore, the present study aimed to investigate the impact of personalized nursing on treatment adherence and clinical symptoms among patients with prostatitis. The intervention was designed to address multiple domains, including baseline assessment, health education, dietary guidance, behavioral modification, and follow-up maintenance. By targeting both medical and lifestyle-related factors, we sought to determine whether personalized nursing could yield meaningful improvements in patient outcomes.

## Materials and methods

### Ethics statement

All patients signed the written informed consent form. The study was approved by the Ethic Committee of The First Affiliated Hospital of Harbin Medical University.

### Study subjects

A total of 85 prostatitis patients admitted to the Department of Urology at The First Affiliated Hospital of Harbin Medical University from August 2019 to September 2020 were collected and randomly grouped into two groups. The control group (*n* = 47) adopted the conventional nursing mode, with an average age of 42.26 ± 4.35 years old. The observation group (*n* = 38) adopted personalized nursing mode on top of the control group, with a mean age of 41.00 ± 3.78 years old.

### Inclusion and exclusion criteria

Inclusion criteria: patients met the diagnostic criteria of type III prostatitis in the Chinese urology and andrology disease diagnosis and treatment guidelines, with a disease duration of > 3 months, with pain in the perineum, suprapubic region, and lower back, symptoms such as urinary frequency and urgency, increased nocturia, and waiting for urination, and the routine examination of the prostate fluids showing the count of leukocytes ≥ 10/HP and reduced number of lecithin bodies; those who had not undergone any other previous treatments. Exclusion criteria: those with a history of urethra and prostate surgery; those with prostatitis in combination with other diseases; those allergic to drugs employed in this study; those with heart, liver, kidney and coagulation dysfunction; those combined with severe cardiovascular and cerebrovascular diseases, hematopoietic diseases, liver and kidney dysfunction, or other severe endocrine system diseases.

### Nursing methods

Both groups received tamsulosin hydrochloride sustained-release capsules (Shenzhen Wanhe Pharmaceutical Co., Ltd., approval number H20223698) at a dose of 0.2 mg once daily, with each treatment cycle lasting 30 days.

The control group was managed with routine nursing care. Patients were instructed to maintain a light diet, abstain from smoking and alcohol, and engage in regular physical exercise during treatment. They were advised to avoid prolonged sitting and urinary retention, keep the perineal area dry and clean, and adhere to proper personal hygiene. Health education was provided regarding basic disease knowledge and the importance of nursing interventions, and patients’ questions were addressed patiently. They were encouraged to maintain a regular lifestyle, ensure adequate rest, and adopt a positive mood. Good hygiene practices were reinforced, including frequent changing of clothing and daily warm sitz baths before bedtime (water temperature 40–42 °C, duration 15 min). Sexual activity was prohibited during treatment. Additionally, telephone follow-ups were conducted once a week at fixed times.

Patients in the observation group adopted personalized nursing model on top of the control group.

Basic information assessment: After the patients were admitted to the hospital, a preliminary survey of their condition, psychological state, and behavior was conducted by the healthcare provider to further assess their compliance and acceptance of nursing. Nursing strategies tailored to the individual patient were developed based on the patient’s individual assessment.

Information intervention: Healthcare staffs made questionnaires by consultation with clinical experts in the hospital and reference to relevant literature, and conducted the survey to understand the patients’ cognition of prostatitis, and according to the results, the staffs carried out appropriate educational activities, including precautions and self-management and other contents. Healthcare professionals need to take the initiative to communicate with patients to understand their true thoughts about the treatment of the disease, answer their questions, and encourage them to fully understand the negative impact of bad mood on treatment and take this opportunity to enhance patients’ confidence in treatment.

Dietary intervention: A structured one-on-one interview approach was adopted, using the *Patient Behavior and Self-Management Assessment Scale* to systematically identify issues in emotional regulation, dietary habits, medication adherence, and exercise routines. For patients with insufficient dietary management, influencing factors were carefully documented, including family cooking practices, individual food preferences, and misconceptions regarding dietary restrictions related to the disease. Based on each patient’s specific situation, nurses developed quantifiable dietary guidance plans, such as ensuring a daily intake of 500 g of vegetables and 200 g of fruit. The goal was to achieve balanced nutrition, thereby improving immune function and enhancing resistance to disease.

Behavior intervention: Interventions were implemented to modify inappropriate behaviors in the domains of emotion, medication, diet, exercise, clothing, and other daily habits. For emotional management, patients received a 15–20 min communication session with nurses once per week, during which targeted reassurance and encouragement were provided. Family members were guided to participate in support, such as engaging in 30 min of daily light conversation with the patient. Patients were also recommended specific coping strategies, including reading prose, listening to relaxing music, or attending community board game activities. For medication management, a detailed drug list was prepared, indicating administration timing (before or after meals), dosage, and treatment duration. Patients were instructed to establish reminders, either via mobile phone alarms or through family members at mealtimes. It was emphasized that any changes in medication must follow physicians’ prescriptions and not be made independently. For dietary management, patients were required to follow individualized dietary guidance. Weekly follow-ups were conducted via WeChat to monitor adherence and make timely adjustments based on feedback. For exercise management, activities were tailored to physical condition. Younger patients were advised to jog daily for 30 min (at 6–7 km/h pace), middle-aged patients to swim 2–3 times per week (40 min each session), and older patients to perform health exercises such as Baduanjin once daily for 15 min. Patients were asked to maintain exercise logs. For clothing, patients were instructed to wear loose cotton underwear, change daily, and avoid restrictive garments such as tight jeans, in order to reduce perineal temperature. For other behavioral management, a prohibition checklist was developed. Patients were advised to avoid sitting longer than 1 h (stand and move every 40 min), limit cycling to no more than 30 min per session, urinate every 2–3 h, and maintain regular bowel movements (1–2 times daily). Guidance was also provided on proper prostate massage (twice weekly, 5 min per session, with gentle pressing from both sides toward the central sulcus) and on warm sitz baths (water temperature 40–42 °C, once daily for 15 min).

Maintenance intervention: A standardized follow-up mechanism was established. Nurses conducted telephone follow-ups once per week at fixed times, with each call lasting at least 10 min. Patients’ adherence to diet, exercise, and medication was assessed using the “Health Behavior Maintenance Evaluation Form.” In addition, face-to-face outpatient follow-ups were arranged once per month. During these visits, patient behavior records and physical condition were reviewed, and tailored solutions were provided for specific problems. For example, if a patient had difficulty maintaining regular exercise, the program was adjusted to a more feasible option such as 30 min of daily walking. Family members were encouraged to participate throughout the process by accompanying patients to outpatient visits and recording their daily behaviors. Patients who demonstrated good maintenance of healthy behaviors received verbal praise and small incentive gifts (e.g., a water cup) during follow-ups, while those with poor adherence were given an analysis of barriers and a revised intervention plan to ensure feasibility and sustainability.

Both groups received interventions for one month.

### Observation indicators

1. The NIH-CPSI was implemented to assess prostate symptoms before and after nursing in the two groups. The assessment was divided into four parts: pain or discomfort symptoms for 0–21 points, urinary symptoms for 0–10 points, symptom impact for 0–6 points, and quality of life for 0–6 points, and the higher the scores of each part, the more serious the condition was.
2. Self-rating Anxiety Scale (SAS) and self-rating Depression Scale (SDS) were utilized to assess the patients’ psychological status, with a full score of 100 points, and higher scores indicated more severe anxiety or depression in the patients.
3. Prior to and after nursing, patients held their urine in advance and drank 500–1,000 mL of water, and after generating the maximum desire to urinate, patients were instructed to urinate naturally into the urinary catheter, and the average and maximum urinary flow rates were calculated at the end of the examination.
4. The Self-Rated Abilities for Health Practices Scale (SRAHP) was employed to assess the health behaviors of patients in the two groups prior to and after nursing. The scale included four dimensions, including health responsibility (7 items), nutrition (8 items), exercise (7 items), and psychological well-being (6 items), and each item was scored from 0 to 4 points, with higher scores indicating better health behavior.
5. Prostatic fluid samples were collected from patients in both groups before and after nursing intervention. Prior to collection, patients were instructed to empty the bladder and abstain from sexual activity for three days. With the patient in the knee-chest position, the clinician wore sterile disposable gloves and applied saline for finger lubrication. The prostate was palpated transrectally, and gentle massage was performed from the lateral lobes toward the central sulcus, with pressure limited to avoid significant discomfort. The first drop of prostatic fluid was collected into a sterile centrifuge tube, ensuring a minimum volume of ≥ 0.3 mL. Immediately after collection, samples were placed at 4 °C and transported to the laboratory within 30 min. Centrifugation was performed at 3000 r/min (radius 8 cm) for 10 min, and the supernatant was aliquoted into two sterile EP tubes (0.1–0.15 mL each) and stored at −80 °C until analysis, avoiding repeated freeze–thaw cycles. Levels of tumor necrosis factor-*α* (TNF-α), prostaglandin E2 (PGE_2_), and cyclooxygenase-2 (COX-2) were quantified using enzyme-linked immunosorbent assay (ELISA) kits (Shanghai Enzyme-linked Biotechnology Co., Ltd., China; catalog numbers: TNF-α, ml106471; PGE_2_, ml057929; COX-2, ml062904).
6. Treatment adherence between the two groups after nursing were compared. The adherence standards were formulated based on whether the patients can actively accept regular treatment or not: patients actively accepted regular treatment without nurse supervision for full adherence; patients actively cooperated with regular treatment after nurse supervision for partial adherence; those incorporated with regular treatment after nurse supervision for non-adherence. Adherence rate = (number of full adherence cases + number of partial adherence cases)/total number of cases × 100%.

### Statistical analysis

GraphPad Prism 8.0 software (Graph Pad Inc., La Jolla, CA, USA) was applied to process the data. Measurement data were depicted as mean ± standard deviation ( $\bar{x}$ ± SD) and analyzed by the t test. Numeration data were depicted as *n* (%) and analyzed by the chi-square test. Differences were considered statistically significant when *p* < 0.05.

## Results

### NIH-CPSI scores before and after nursing

There was no difference in pain, dysuria, impact of symptoms and quality of life scores between the two groups prior to nursing (*p* > 0.05); after nursing, the relevant scores were diminished, and the scores of the observation group were all lower versus the control group (*p* < 0.05) (Table 1).

**Table 1:** NIH-CPSI scores before and after nursing between the two groups.

NIH-CPSI scores	Time	Control group (*n* = 47)	Observation group (*n* = 38)	*P*
Pain	Before nursing	16.26 ± 2.09	15.79 ± 2.21	0.318
	After nursing	10.40 ± 1.16*	6.08 ± 1.36*	<0.001
Dysuria	Before nursing	7.60 ± 1.42	7.95 ± 1.56	0.283
	After nursing	6.28 ± 1.32*	3.97 ± 1.14*	<0.001
Impact of symptoms	Before nursing	5.45 ± 0.58	5.26 ± 0.75	0.191
	After nursing	4.15 ± 0.80*	2.95 ± 0.51*	<0.001
Quality of life	Before nursing	5.06 ± 0.78	5.08 ± 0.81	0.908
	After nursing	4.30 ± 0.50*	3.08 ± 0.48*	<0.001

*P < 0.05 vs the same group before nursing.

### Negative emotion scores before and after nursing

Prior to and after nursing, the SDS scores in the control group were 62.38 ± 5.86 and 37.15 ± 4.17, respectively, and the SDS scores in the observation group were 63.13 ± 6.28 and 30.18 ± 3.10, respectively; the SAS scores in the control group were 55.77 ± 5.72 and 32.43 ± 4.64, respectively, and the SAS scores in the observation group were 54.24 ± 5.32 and 28.11 ± 3.80, respectively. No difference was found in SDS and SAS scores between the two groups prior to nursing (*p* > 0.05); the relevant scores were reduced after nursing, and the scores of the observation group were all lower versus the control group (*p* < 0.05) (Table 2).

**Table 2:** Negative emotion scores before and after nursing between the two groups.

Negative emotion scores	Time	Control group (*n* = 47)	Observation group (*n* = 38)	*P*
SDS scores	Before nursing	62.38 ± 5.86	63.13 ± 6.28	0.571
	After nursing	37.15 ± 4.17*	30.18 ± 3.10*	<0.001
SAS scores	Before nursing	55.77 ± 5.72	54.24 ± 5.32	0.210
	After nursing	32.43 ± 4.64*	28.11 ± 3.80*	<0.001

*P < 0.05 vs the same group before nursing.

### Urine flow rate before and after nursing

Prior to and after nursing, the maximum urine flow rate in the control group was 12.77 ± 1.06 and 17.45 ± 1.22; the maximum urine flow rate in the observation group was 13.02 ± 1.20 and 18.82 ± 1.35; the average urine flow rate in the control group was 9.15 ± 0.77 and 10.38 ± 1.04; the average urine flow rate in the observation group was 9.21 ± 0.92, and 10.92 ± 0.90. There was no difference between the two groups in terms of the maximum urine flow rate and average urinary flow rate before nursing (*p* > 0.05); they were improved after nursing, and those in the observation group were higher versus those in the control group (*p* < 0.05) (Table 3).

**Table 3:** Urine flow rate before and after nursing between the two groups.

Urine flow rate	Time	Control group (*n* = 47)	Observation group (*n* = 38)	*P*
Maximum urine flow rate	Before nursing	12.77 ± 1.06	13.02 ± 1.20	0.311
	After nursing	17.45 ± 1.22*	18.82 ± 1.35*	<0.001
Average urine flow rate	Before nursing	9.15 ± 0.77	9.21 ± 0.92	0.744
	After nursing	10.38 ± 1.04*	10.92 ± 0.90*	0.014

*P < 0.05 vs the same group before nursing.

### Health behaviors before and after nursing

No significant difference was noted in the scores of health responsibility, nutrition, exercise and psychological well-being between the two groups prior to nursing (*p* > 0.05); the relevant scores were raised after nursing, and those scores in the observation group were higher versus the control group (*p* < 0.05) (Table 4).

**Table 4:** Health behaviors before and after nursing between the two groups.

Health behaviors	Time	Control group (*n* = 47)	Observation group (*n* = 38)	*P*
Health responsibility	Before nursing	17.00 ± 4.87	17.79 ± 4.26	0.434
	After nursing	21.26 ± 3.02*	25.66 ± 2.53*	<0.001
Nutrition	Before nursing	19.30 ± 3.62	19.42 ± 3.17	0.873
	After nursing	22.32 ± 2.76*	26.39 ± 2.02*	<0.001
Exercise	Before nursing	18.51 ± 3.58	18.16 ± 3.62	0.657
	After nursing	20.38 ± 2.69*	25.61 ± 1.33*	<0.001
Psychological well-being	Before nursing	16.04 ± 3.40	16.87 ± 3.14	0.250
	After nursing	19.51 ± 2.33*	22.58 ± 1.48*	<0.001

*P < 0.05 vs the same group before nursing.

### Inflammatory factor levels before and after nursing

There was no difference in TNF-*α*, PGE_2_ and COX-2 levels between the two groups before nursing (*p* > 0.05); the relevant levels were decreased after nursing, and those in the observation group were lower than those in the control group (*p* < 0.05) (Table 5).

**Table 5:** Inflammatory factor levels before and after nursing between the two groups.

Inflammatory factor	Time	Control group (*n* = 47)	Observation group (*n* = 38)	*P*
TNF-α (pg/mL)	Before nursing	25.21 ± 4.30	26.03 ± 4.21	0.380
	After nursing	18.63 ± 3.23	16.38 ± 3.47	0.003
PGE_2_ (pg/mL)	Before nursing	25.63 ± 5.11	24.78 ± 4.41	0.420
	After nursing	17.31 ± 3.09	14.09 ± 2.31	<0.001
COX-2 (pg/mL)	Before nursing	16.55 ± 3.02	16.28 ± 2.23	0.648
	After nursing	10.67 ± 2.09	7.14 ± 1.59	<0.001

### Treatment adherence after nursing

After nursing, the adherence rate of the control group was 74.47%, and the rate of the observation group was 94.74%. The treatment adherence rate of the observation group after nursing was higher in contrast to the control group (*p* < 0.05) (Table 6).

**Table 6:** Treatment adherence after nursing between the two groups.

Treatment adherence	Control group (*n* = 47)	Observation group (*n* = 38)	*P*
Full adherence	22 (46.81)	32 (84.21)	-
Partial adherence	13 (27.66)	4 (10.53)	-
Non-adherence	12 (25.53)	2 (5.26)	-
Adherence rate	35 (74.47)	36 (94.74)	0.012

## Discussion

Prostatitis, commonly seen in urology departments (20), is an inflammatory disorder of prostate gland and can affect 2–16% of men around the world (21). This paper focused on the effects of personalized nursing on treatment adherence and clinical symptoms in patients with prostatitis. Currently, clinical evidence regarding the role of personalized nursing in prostatitis is limited, and our study provides preliminary exploration in this area.

As previously reported, NIH-CPSI is developed to accurately measure the urinary symptoms, pain, and quality of life relevant to CP/CPPS (22). NIH-CPSI scores are considered as a validated measure widely implemented to assess the symptoms of CP/CPPS (23). Furthermore, the NIH-CPSI total scores are regarded as reliable, valid, responsive measures of prostatitis symptoms in patients who need primary and secondary care (24). A previous study has demonstrated that anxiety and depression can play a significant role in CP/CPPS pathogenesis, development and prognosis (25). It is also reported that psychological care is involved in the enhancement of therapeutic effects on type IIIB prostatitis, the relief of prostatitis pain, anxiety and depression, and the recovery of prostatic function. After the intervention, the total effectiveness rate is higher and the NIH-CPSI, SAS and SDS scores are remarkably elevated (26). Our findings are consistent with these reports, as personalized nursing reduced NIH-CPSI, SAS, and SDS scores. Our findings may be interpreted in light of the Health Belief Model, suggesting that individualized psychological support and health education could improve patients’ perceptions of illness, enhance adherence, and thereby facilitate recovery. Although specific studies on personalized nursing in prostatitis are scarce, evidence from nurse-led psycho-educational interventions in prostate cancer suggests similar benefits, such as improved self-management and psychological well-being in prostate cancer survivors undergoing nurse-led interventions (27).

The urination pattern and maximum velocity are employed as a reference to measure the health condition of prostate and bladder (28). Precise nursing service, which included psychological counseling and post-hospital discharge care, has been shown to raise maximum urinary flow rate and reduce postoperative urinary incontinence, thereby improving rehabilitation and quality of life (29). Our study findings corroborate these, with higher maximum and average urinary flow rates, better health behavior scores (including health responsibility, nutrition, exercise, and psychological well-being), and reduced levels of inflammatory markers (TNF-*α*, PGE_2_, COX-2) in the personalized nursing group versus control. These results align with broader evidence showing that nurse-delivered interventions enhance outcomes in prostate-related conditions. For instance, Mao et al. demonstrated that continuity nursing based on the Knowledge-Attitude-Practice model significantly improved self-efficacy, treatment compliance, and quality of life in elderly patients with benign prostatic hyperplasia (30).

Treatment adherence can be regarded as the degree to which patients’ behaviors are consonant with health or medical advice them receive as part of their treatment regimen (31). In our study, adherence was significantly higher in the personalized nursing group (94.74%) compared to the control group (74.47%). This enhancement mirrors findings in other nurse-led models, such as automated personalized text messaging improving adherence in prostate cancer screening (32). Additionally, nurse-led supportive or psycho-educational care has demonstrated positive impacts on survivors’ engagement and adherence to care plans (14).

In summary, our study suggests that personalized nursing has potential benefits in improving adherence, health behaviors, and quality of life in patients with prostatitis. This study provides a preliminary foundation for further exploration of the role of personalized nursing in this population. However, several limitations should be acknowledged. First, the sample size was relatively small, and the study was conducted at a single center, which limits the representativeness and generalizability of the findings. Second, the cross-sectional design restricts causal inference. Third, the follow-up period was short, making it impossible to assess the long-term sustainability of the intervention effects. Fourth, our study did not incorporate an explicit theoretical or conceptual model to guide the intervention design or interpretation of outcomes, which may limit the depth of theoretical explanation.

Despite these limitations, the study has notable strengths. It innovatively applied personalized nursing in prostatitis care, assessed outcomes from multiple dimensions including symptoms, psychological indicators, and inflammatory markers, and employed internationally recognized tools such as NIH-CPSI, enhancing comparability with other studies. Future studies should adopt multi-center, large-sample, and longitudinal designs to validate and expand these findings. Moreover, incorporating established nursing theories or health behavior models may further clarify the mechanisms underlying the effectiveness of personalized nursing and enhance its applicability across different clinical settings.

## Data Availability

The original contributions presented in the study are included in the article/supplementary material, further inquiries can be directed to the corresponding author/s.
